# Kick proficiency and skill adaptability increase from an Australian football small-sided game intervention

**DOI:** 10.3389/fspor.2022.1026935

**Published:** 2022-10-26

**Authors:** Nathan Bonney, Paul Larkin, Kevin Ball

**Affiliations:** Institute for Health and Sport, Victoria University, Melbourne, VIC, Australia

**Keywords:** kicking, skill acquisition, SSG, team sport, training intervention

## Abstract

This investigation is the first to explore the effect of a 4 week small-sided game (SSG) and traditional training intervention on player kick proficiency and player adaptability in Australian football. Twenty-two amateur Australian football players (mean ± SD; age 22.3 ± 2.46; height 182.4 ± 5.25; weight 82.1 ± 6.10; years playing senior amateur football 3.86 ± 3.09) were randomly selected into either a traditional training group (*n* = 11) or a SSG group (*n* = 11). Traditional training involved activities where skills were generally executed in isolation and with minimal contact (e.g., kicking lane drill or possession football). The SSG training group participated in 5v6 competitive games on varied shaped areas (approximately 272 m^2^ per player) and changing constraints (e.g., game tempo, game rules). All players participated in the team training sessions; however, the SSG group participated in a 4 × 3min training protocol, with 60 seconds recovery, in the last 20-min of the session. The SSG group participated in these alternative sessions twice a week for 4 weeks. Results indicated only the SSG enhanced their kick proficiency (17%) and were found to be more adaptable. The intervention group executed more kicks over longer distances (i.e., 20–40 m), made quicker decisions (e.g., executing more kicks in < 1s), applied more pressure to the opposition when they were executing a skill and were more likely to “take the game on” by decreasing the amount of times a skill was executed from a stationary position. The results of this study can be used by coaches when designing and implementing training programs as different training strategies will elicit different player behavioral adaptations.

## Introduction

Australian football is an open skilled invasion team sport requiring players to constantly adapt their performance based upon the interactions of their teammates and the opposition. Successful players and teams possess high levels of both technical skill proficiency (1, 2) and physiological capacities (3) under varying match-play conditions (4). Robertson et al. (2) found teams who were able to obtain greater ball possessions and maintain greater kicking proficiency than their opposition were able to influence the match outcome to a greater extent. Furthermore, Bonney et al. (5) found kick proficiency, assessed within a field-based small-sided game assessment (SSG), was 97% successful in identifying players as either novice or sub-elite. The implementation of appropriate training methodologies is therefore an important consideration to ensure player technical skill is developed in the most effective and efficient manner.

Contemporary Australian football research has provided greater insights into training constructs in an attempt to better prepare players for match-play conditions (6–8). In particular, exposing players to decision-making interventions has been suggested to improve player performance. Clemente et al. (9) found technical executions to be associated with tactical decision-making. Decision-making programs (e.g., video-based, skill-based) can develop a players ability to better understand the game enabling the player to solve tactical situations and execute more proficient technical decisions during competition (10, 11). However, further research is required to outline the effectiveness of training interventions at enhancing player match-play kicking proficiency and to become more adaptable during match-play performance. Such information is critical for sport coaches so they can more effectively design training interventions to prepare players for the uncertain nature of match-play.

Skill adaptability is an individual's ability to adjust their performance based on the changing stimulus within the performance environment (12). Researchers have noted how coaches should attempt to develop adaptive behaviors of players, through multiple learning experiences, to enhance the sensorimotor adaptive processes so players can make decisions and perform actions to achieve the task goal (13, 14). In Australian football, the ability to be adaptable to the dynamic performance context is critically important. Players must perceive and interpret their environment (e.g., understand where the ball is, where their opponents and teammates are, where the space is) and then act upon this perception (e.g., lead for the ball by running toward the open space). A practical example of this may include a player taking possession of the ball, quickly canvassing the environment to identify an appropriate teammate to kick the ball to before being tackled. The player may select a teammate to kick the ball to but if their selection and action take too long (e.g., >1s) this option may disappear and another opportunity may arise (e.g., a handball to another teammate running past). The development of tasks, where players are required to be more adaptable, may enhance individual and team competence which may lead to greater team success.

Small-sided games (e.g., unpredictable modified games, using adapted rules and playing areas to achieve specific performance outcomes), in comparison to traditional training (e.g., predictable and repetitious drills with limited decision making) have been used in team sport as a way of enhancing player decision making (15), technical and perceptual abilities (16) and physical capabilities (17). More recently SSGs have been manipulated to produce specific technical and physical performances (7) and used as a tool to identify players as either higher or lesser skilled (5, 18). For example, by reducing the number of players and area size the total number of technical actions per player increases or by limiting the number of ball contacts more shots at goal and faster playing patterns may occur (19). The majority of this research has focused on acute responses, however; SSGs have also been suggested to be an important teaching approach when applied from a long-term perspective (i.e., intervention) (9). Considering the versatility of SSGs it would be interesting to evaluate their effectiveness in developing a player's kicking proficiency and adaptability and if so how this response compares to traditional training methods (e.g., activities where skills are repetitive and generally executed in isolation, with minimal contact, such as kicking lane drills).

The assessment of training interventions on player kick proficiency and adaptability in Australian football has not been researched. Such investigations would provide critical understandings to coaches regarding the design and implementation of effective training programs. Overall, this study is investigating if the implementation of Australian football SSGs can develop a players kicking proficiency and playing adaptability greater than traditional training drills.

## Methods

### Participants

To estimate the adequate sample size for the investigation a priori analysis was conducted with G^*^Power 3.1 (20, 21). An analysis of variance with an effect size of 0.80 and alpha level of 0.05 was used. Results indicated a total of a 19 participants (approximately 10 in each group) was required to achieve a power of 0.90.

Twenty-two amateur Australian football players (i.e., community-based players who typically participate in two team training sessions per week and compete in one match on the weekend) participated in the study. The players (mean ± SD; age 22.3 ± 2.46; height 182.4 ± 5.25; weight 82.1 ± 6.10; years playing senior amateur football 3.86 ± 3.09) were randomly selected into either an intervention (“traditional”) group (*n* = 11) or a control (“SSG”) group (*n* = 11). All players were injury free at the time of testing. The project was approved by the University Human Research Ethics Committee (HRE21-065) and all players gave written informed consent before participating in the study.

### Procedures

The Australian football 5v6 small-sided game was used to conduct the pre and post-test assessment on the 22 players. Previous research has found this assessment to be a valid and reliable assessment of Australian football kicking proficiency (ICC ± 95% CL = 0.82 ± 0.45–0.94) (5). Both the traditional and SSG groups participated in the initial testing session 6 weeks prior to the first practice match of the 2021 season. All players participated in the team training sessions; however, the SSG group participated in a separate training protocol in the last 20-min of the session (whilst the traditional training group continued their normal training session for the final 20-min). Traditional training methods involved activities where skills were generally executed in isolation and with minimal contact (e.g., kicking lane drill or possession football). Players found these types of activities more predictable and easier to attain successful outcomes. The SSG group participated in these alternative sessions twice a week for 4 weeks. Each SSG was approximately 3,000 m^2^ (approximately 272 m^2^ per player) and varied in shape. For example, week 2 involved players participating in the 50m area, playing across the ground; week 3 was played on a rectangular area and required players to mark the ball within a circle to score a point; week 4 was played in the 50m area and required players to kick the ball immediately after receiving it and involved a 6v6 contest (an extra player was recruited for this 1 week) and in week 5 (played in a square area formation) the game tempo was constantly changed where players had to slow down and speed up play in response to a whistle blast. The SSG group participated in these activities for 4 × 3 min quarters with 60s recovery. Week six involved post testing of both the traditional and SSG training groups.

To ensure stabilization of the pre-test data, all players participated in a familiarization session for approximately 5 min before the start of the pre-test, as recommended by Currell and Jeukendrup (22). A 5 min break then occurred before commencement of the test. Both the pre and post-test sessions were conducted on the team's regular training ground, during the pre-season (late summer) where the weather is fairly consistent and dry.

During the test, three cameras were positioned on the field to capture the test performance. One camera was positioned five meters behind the goal posts and the other two were positioned on opposite sides of the playing area approximately two meters outside the boundary line.

A 5-point Likert scale questionnaire (i.e., 1 strongly disagree; 2 disagree; 3 neutral; 4 agree; 5 strongly agree) (23) was completed by the players participating in the intervention study and the two coaches 10 min after completion of the post-test. The coaches had an average of 17 ± 11 years coaching experience. One held a Level-2 coaching accreditation and the other held a Level-3 coaching accreditation. The players and coaches completed separate questionnaires. The player questionnaire had eight questions pertaining to match specificity, competition preparation, skill enhancement, adaptability, enjoyment, physical demand, mental demand and tactical awareness. The coach questionnaire contained four questions pertaining to player competition preparedness, player tactical awareness, player match-play skill proficiency and player adaptability.

### Data analysis

Video footage from three cameras were stacked (i.e., having the three camera angles showing on the one screen side-by-side) then coded using a custom made Microsoft Excel workbook (24). The variables selected (and their assessment) are the same as those applied by Bonney et al. (5). Variables assessed were kick leg (dominant, non-dominant); kick score [1–5]; handball rating (positive, neutral, negative), kick distance (0–20 m, 20–40 m, 40 m+), time before kick or handball execution (<1s, 1–2s, 2–4s, 4+s); pressure when executing a skill (pressure, no pressure); locomotor movement when executing a skill (stationary, run); receiving player locomotor movement (leading and covered, leading and open, stationary and covered, stationary and open). The variable definitions are presented in Table 1.

**Table 1:** Criteria to code variables.

**Variable**	**Sub-variable**	**Definition**
Kick leg	Dominant	The kick was executed with the player's dominant leg
	Non-dominant	The kick was executed with the player's non-dominant leg
Kick proficiency	1–5	As previously published by Bonney et al. (25)
Handball proficiency	Positive	Handball goes to the advantage of that team
	Neutral	The handball goes to a 50/50 opportunity of re-gaining possession
	Negative	The opposition intercept the ball or the ball does not go to the advantage of that team
Kick distance	0–20m	The kick distance was between 0 and 20m
	20–40m	The kick distance was between 20 and 40m
	40m+	The kick distance was over 40m
Time before skill execution	<1s	The kick or handball was executed in under 1 sec
	1–2s	The kick or handball was executed between 1-2 sec
	2–4s	The kick or handball was executed between 2-4 sec
	4+s	The kick or handball was executed in over 4 sec
Pressure	Pressure	The player with the ball had an opposition player within 3 meters when executing a kick or handball
	No pressure	No opposition player was within 3 meters of the player with the ball when executing a kick or handball
Movement when executing a kick or handball	Stationary	A player with the ball standing still, walking, shuffling or slowly jogging when executing a kick or handball
	Run	Fast jog or sprint
Receiving player movement	Leading and covered	A player leading for the ball has an opposition player within 3 meters
	Leading and open	A player leading for the ball has no opposition player within 3 meters
	Stationary and covered	The player receiving the ball is either stationary, walking, shuffling or slowly jogging and has an opposition player within 3 meters
	Stationary and open	The player receiving the ball is either stationary, walking, shuffling or slowly jogging with no opposition player within 3 meters

Both the pre and post-test performances were assessed by the same experienced observer, who has assessed more than 80 h of Australian football footage. Coding reliability was assessed using an intra-class correlation coefficient (3,k) (26). The intra-class correlation coefficient parameters used were poor (<0.5), moderate (0.5–0.75), good (0.75–0.90) and excellent (>0.90) (26). The result indicated an excellent level of reliability (r = 0.96–0.99).

A two-way repeated measures analysis of variance (ANOVA) was used to analyze the effect of intra-factor (pre-post) and inter-factor (group) of the SSG and traditional training groups (27). Assumptions of normality for the variables were analyzed using the Shapiro-Wilk test. Mean and standard deviation were calculated for each variable. The alpha level of statistical significance was set at *p* < 0.05. The magnitude of the independent variables were calculated using partial eta squared with effect sizes classified as small ($\eta_{\text{p}}^{2}$ = 0.01), medium (η_p_^2^ = 0.06) and large ($\eta_{\text{p}}^{2}$ = 0.14) (28, 29). *Post hoc* significance was calculated using Cohen's *d* (30). Effect sizes (d) were classified as trivial (0 to 0.19), small (>0.20 to 0.49), medium (>0.50 to 0.79) and large (>0.80). All statistical analysis were conducted using the free and open-source program JASP version 0.16 (31).

The mean and standard deviation for the Likert scale questions were calculated and classified using previously published descriptors by Bonney et al. (25) strongly disagree (1–1.9), disagree (2–2.9), agree (3–3.9), strongly agree (4–4.9).

## Results

### Kick proficiency

A two-way ANOVA was conducted to examine the effect of a traditional and a SSG training intervention on kick proficiency. A significant interaction was found between the effects of the traditional and SSG training intervention on kick proficiency, *F*_(1,40)_ = 4.24, *p* = 0.05, η_p_^2^ = 0.10. *Post hoc* analysis showed the intervention group significantly improved their kicking proficiency with a large effect size (*p* = <0.01, *d* = 1.47), but a significant difference was not found for the control group (*p* = 0.95) (Table 2).

**Table 2:** Mean ± SD for pre-test and post-test variables.

	**Pre intervention**		**Post intervention**										
	**Mean ±SD**	**95% CI**	**Mean ±SD**	**95% CI**	**Mean difference**	**95% CI**	**SE**	**t**	**p**		**Cohen's d (95% CI)**	**95% CI**	**Effect size**
**Kick proficiency**													
Traditional group	56.87 ± 16.61	44.99–68.75	59.50 ± 11.75	52.04–66.96	2.64	−10.86–16.13	5.03	0.52	0.95		0.22	−0.97–1.42	
SSG group	56.20 ± 7.26	51.32–61.08	73.47 ± 10.00	66.75–80.18	17.26	3.83–30.70	5.01	3.44	<0.01	**	1.47	0.20–2.74	Large
**Kicking leg**													
Dominant–traditional group	8.64 ± 5.18	5.16–12.12	8.55 ± 5.26	5.01–12.08	−0.18	−4.79–4.43	1.72	−0.05	1.00		0.02	−1.16–1.21	
Dominant–SSG group	8.64 ± 2.38	7.04–10.23	8.73 ± 2.20	7.25–10.20	0.09	−4.52–4.70	1.72	0.05	1.00		0.02	−1.16–1.21	
Non-dominant–traditional group	0.18 ± 0.60	−0.24–0.61	0.18 ± 0.41	−0.24–0.61	2.50	−0.80–0.80	0.30	8.40	1.00		0.00	−1.18–1.18	
Non-dominant–SSG group	0.82 ± 0.98	0.39–1.24	0.64 ± 0.67	0.21–1.06	−0.18	−0.98–0.62	0.30	−0.61	0.93		0.26	0.93–1.45	
**Handball proficiency rating**													
Positive–traditional group	1.18 ± 1.17	0.53–1.84	1.00 ± 0.78	0.35–1.66	−0.18	−1.41–1.05	0.46	−0.40	0.98		0.17	−1.35–1.02	
Positive–SSG group	1.09 ± 1.04	0.44–1.75	0.82 ± 1.25	0.16–1.47	−0.27	−1.50–0.96	0.46	−0.60	0.93		−0.25	−1.44–0.93	
Neutral–traditional group	0.64 ± 0.67	0.31–0.97	0.46 ± 0.69	0.12–0.79	−0.18	−0.80–0.44	0.23	−0.78	0.86		−0.33	−1.52–0.85	
Neutral–SSG group	0.09 ± 0.30	−0.24–0.42	0.18 ± 0.41	−0.15–0.51	0.09	−0.53–0.71	0.23	0.39	0.98		0.17	−1.02–1.35	
Negative–traditional group	0.18 ± 0.60	−0.02–0.39	0.00 ± 0.00	−0.21–0.21	−0.18	−0.57–0.20	0.14	−1.27	0.59		−0.54	−1.94–0.86	
Negative–SSG group	0.09 ± 0.30	−0.12–0.30	0.00 ± 0.00	−0.21–0.21	−0.09	−0.48–0.29	0.14	−0.63	0.92		0.27	−1.72–1.18	
**Distance of kick**													
0–20m–traditional group	3.00 ± 1.95	1.89–4.11	3.36 ± 2.01	2.26–4.47	0.36	−1.71–2.44	0.77	0.47	0.97		0.20	−0.99–1.39	
0–20m–SSG group	6.36 ± 2.11	5.26–7.47	2.46–0.93	1.35–3.56	−3.91	−5.98–−1.83	0.77	−5.05	<0.001	***	2.15	0.79–3.51	Large
20–40m–traditional group	5.36 ± 3.85	3.50–7.22	5.18 ± 4.14	3.32–7.04	−0.18	−3.67–3.31	1.30	−0.14	1.00		0.06	−1.12–1.24	
20–40m–SSG group	2.73 ± 1.42	0.87–4.59	6.27 ± 1.79	4.41–8.13	3.55	0.06–7.03	1.30	2.73	0.05	*	1.16	−0.08–2.40	Large
>40m–traditional group	0.36 ± 0.67	0.08 ± 0.65	0.09 ± 0.30	−0.20 ± 0.38	−0.27	−0.81–0.27	0.20	−1.36	0.53		−0.58	−1.78–0.62	
>40m–SSG group	0.09 ± 0.30	−0.20 ± 0.38	0.36 ± 0.51	0.08 ± 0.65	0.27	−0.27–0.81	0.20	1.36	0.53		0.58	−0.62–1.78	
**Time taken before skill execution**													
<1s–traditional group	1.64 ± 1.29	0.78–2.49	0.82 ± 1.08	−0.04–1.67	−0.82	−2.42–0.79	0.60	−1.34	0.53		−0.58	−1.78–0.62	
<1s–SSG group	0.64 ± 0.81	−0.22–1.49	2.27 ± 2.10	1.42–3.13	1.64	0.03–3.24	0.60	2.73	0.04	*	1.17	−0.07–2.40	Large
1–2s–traditional group	4.27 ± 3.17	2.80–5.75	3.09 ± 2.91	1.62–4.56	−1.18	−3.95–1.58	1.03	−1.15	0.66		−0.49	−1.68–0.71	
1–2s–SSG group	3.00 ± 1.61	1.53–4.47	4.55 ± 1.51	3.07–6.02	1.55	−1.22–4.31	1.03	1.50	0.45		0.64	−0.56–1.84	
2–4s–traditional group	3.64 ± 2.46	2.31–4.96	5.27 ± 2.97	3.95–6.60	1.64	−0.85–4.12	0.93	1.76	0.31		0.75	−0.46–1.96	
2–4s–SSG group	3.00 ± 1.00	1.67–4.33	3.46 ± 1.75	2.13–4.78	0.46	−2.03–2.94	0.93	0.49	0.96		0.21	−0.98–1.39	
>4s–traditional group	1.27 ± 1.10	0.53–2.01	1.64 ± 1.21	0.90–2.38	0.36	−1.03–1.75	0.52	0.70	0.90		0.30	−0.89–1.49	
>4s–SSG group	2.46 ± 1.51	1.71–3.20	0.82 ± 0.98	0.08–1.56	−1.64	−3.03–−0.25	0.52	−3.16	0.02	*	1.35	0.09–2.60	Large
**Pressure**													
Pressure–traditional group	3.27 ± 2.33	1.47–5.07	7.18 ± 5.00	5.38–8.98	3.91	0.53–7.28	1.26	3.11	0.02	*	1.32	0.07–2.58	Large
Pressure–SSG group	2.27 ± 1.74	0.47–4.07	2.55 ± 1.21	0.75–4.35	0.27	−3.10–3.65	1.26	0.220	1.00		0.09	−1.09–1.28	
No pressure–traditional group	7.55 ± 4.74	5.63–9.46	2.91 ± 1.51	1.00–4.82	−4.64	−8.22–−1.05	1.34	−3.47	0.01	**	1.48	−0.21–2.75	Large
No pressure–SSG group	8.36 ± 2.50	6.45–10.28	7.82 ± 2.89	5.91–9.73	−0.55	−4.13–3.04	1.24	−0.41	0.98		0.17	−1.36–1.01	
**Locomotor movement at skill execution**													
Stationary–traditional group	7.55 ± 4.99	5.12–9.97	8.00 ± 5.40	5.58–10.42	0.46	−4.09–5.00	1.69	0.27	0.99		0.11	−1.07–1.30	
Stationary–SSG group	7.73 ± 2.76	5.31–10.15	4.64 ± 1.21	2.22–7.06	−3.09	−7.63–1.45	1.69	−1.82	0.28		−0.78	−1.99–0.43	
Run–traditional group	3.27 ± 2.10	2.05–4.50	2.09 ± 1.51	0.87–3.31	−1.18	−3.47–1.11	0.86	−1.38	0.52		−0.59	−1.79–0.61	
Run–SSG group	2.82 ± 2.14	1.60–4.04	5.73 ± 2.20	4.51–6.95	2.91	0.62–5.2	0.86	3.40	0.01	**	1.45	0.18–2.72	Large
**Delivery to**													
Leading and covered–traditional group	2.36 ± 2.34	1.24–3.49	1.55 ± 1.04	0.42–2.67	−0.82	−2.93–1.30	0.79	−1.04	0.73		−0.44	−1.63–0.75	
Leading and covered–SSG group	3.09 ± 1.58	1.96–4.22	3.36 ± 2.16	2.24–4.49	0.27	−1.84–2.39	0.79	0.35	0.99		0.15	−1.04–1.33	
Leading and open–traditional group	3.27 ± 3.29	1.98–4.57	3.09 ± 2.07	1.80–4.38	−0.18	−2.61–2.24	0.91	−0.20	1.00		−0.09	−1.27–1.10	
Leading and open–SSG group	3.36 ± 0.81	2.07–4.66	3.64 ± 1.50	2.34–4.93	0.27	−2.15–2.70	0.91	0.30	0.99		0.13	−1.06–1.31	
Stationary and covered–traditional group	1.09 ± 1.45	0.14–2.05	2.18 ± 2.36	1.23–3.14	1.09	−0.70–2.88	0.67	1.63	0.37		0.70	−0.51–1.90	
Stationary and covered–SSG group	1.00 ± 1.27	0.05–1.96	0.82 ± 0.75	−0.14–1.77	−0.18	−1.97–1.61	0.67	−0.27	0.99		0.06	−1.13–1.24	
Stationary and open–traditional group	3.73 ± 1.68	2.47–4.99	3.18 ± 2.68	1.92–4.44	−0.55	−2.91–1.82	0.88	−0.62	0.93		−0.26	−1.45–0.92	
Stationary and open–SSG group	2.91 ± 2.17	1.65–4.17	2.55 ± 1.57	1.28–3.81	−0.36	−2.73–2.00	0.88	−0.41	0.98		−0.18	−1.36–1.01	

^*^ indicates *p* = <0.05, ^**^ indicates *p* = <0.01, ^***^ indicates *p* = <0.001, SD, Standard Deviation; CI, Confidence Interval; SE, Standard Error.

### Kicking distance

There was a significant interaction between the effects of the traditional and SSG training intervention on the distances kicks were executed between 0–20m, *F*_(1,40)_ = 15.23, *p* = <0.001, η_p_^2^ = 0.28 and 20–40 m, *F*_(1,40)_ = 4.10, *p* = 0.05, η_p_^2^ = 0.09. *Post hoc* analysis showed the intervention group significantly decreased the amount of times they executed a kick between 0–20m with a large effect size (*p* = <0.001, *d* = 2.15) and significantly increased the amount of times they executed a kick between 20–40 m with a large effect size (*p* = <0.05, *d* = 1.16).

### Time before skill execution

A significant interaction was found between the effects of the traditional and SSG training intervention on the time taken before a skill was executed <1s, *F*_(1,40)_ = 8.40, *p* = 0.01, η_p_^2^ = 0.17 and >4s *F*_(1,40)_ = 7.45, *p* = 0.01, η_p_^2^ = 0.16. *Post hoc* analysis showed the intervention group significantly increased the amount of times they executed a skill in <1s with a large effect size (*p* = <0.05, *d* = 1.17) and decreased the amount of times they executed a skill >4s with a large effect size (*p* = <0.05, *d* = 1.35).

### Pressure when executing a skill

A significant interaction was found between the effect of the traditional and SSG training interventions on the pressure applied to players executing a skill *F*_(1,40)_ = 4.17, *p* = 0.05, η_p_^2^ = 0.09 and non-pressure applied to players executing a skill *F*_(1,40)_ = 4.68, *p* = 0.04, η_p_^2^ = 0.11. *Post hoc* analysis showed the traditional group had an increase in pressure applied to them post the intervention with a large effect size (*p* = <0.05, *d* = 1.32) and a decrease in no-pressure when executing a skill post intervention with a large effect size (*p* = <0.01, *d* = 1.48).

### Locomotor movement at skill execution

A significant interaction was found between the effects of the traditional and SSG training interventions on the locomotor movement pattern at skill execution on the run *F*_(1,40)_ = 11.44, *p* = <0.01, η_p_^2^ = 0.22 but not when executing a skill from a stationary position (*p* = 0.15). *Post hoc* analysis showed only the SSG group increased the amount of times they executed a skill on the run post intervention with a large effect size (*p* = <0.01, *d* = 1.45).

Accordingly to the questionnaire results, the players strongly agreed the SSGs were more specific to match play (4.29 ± 0.45) and prepared players better for competition (4.43 ± 0.62) than traditional training methods. The players (4.29 ± 0.59) and coaches (4.50 ± 0.50) also strongly agreed the SSGs enhanced player skill proficiency greater than traditional training methods and the coaches agreed the players were technically more prepared for competition (4.50 ± 0.50). The players (4.21 ± 0.56) and coaches (4.5 ± 0.50) also strongly agreed the SSGs enhanced player ability to adapt during match-play more than traditional methods.

The players agreed the SSG intervention was physically more challenging (3.93 ± 0.70) and strongly agreed it was more mentally challenging (4.00 ± 0.93) than traditional methods. The players (4.50 ± 0.50) and coaches (4.00 ± 0.00) both strongly agreed the SSG enhanced player tactical awareness more than traditional training methods.

The players strongly agreed the SSG intervention was a more enjoyable way of training (4.57 ± 0.62) in comparison to traditional methods.

## Discussion

This study investigated the differences in player kicking proficiency and player adaptability between traditional and SSG training interventions. After 4 weeks of training only the SSG group enhanced their kicking proficiency and became more adaptable. The post-test results found the SSG group executed more kicks over longer distances (i.e., 20–40m), made quicker decisions (e.g., executing more kicks in <1s), applied more pressure to the opposition when they were executing a skill and were more likely to “take the game on” by decreasing the amount of times a skill was executed from a stationary position. The traditional training group did not significantly improve in any of the variables analyzed.

Overall, the players found the SSG training to be more match specific which helped them to prepare better for match-play. In comparison to traditional training, the players found this type of training enhanced their adaptability whilst also increasing their technical and tactical capabilities. Traditional training generally involves an activity where skills are executed to a pre-determined location and executed predominantly in isolation. For example, a kicking lane drill involves players running and kicking/handballing the ball, in a linear motion, toward the intended target under little or no pressure. Another traditional activity is possession football. This activity involves players maintaining possession of the ball, within a designated area, for a set time. The attacker and defender numbers may vary depending on the goal of the activity (7) with light contact occasionally occurring. Both of these tasks involve little demand from the tactical, psychological and, at times, physiological components. Consequently, these activities distance themselves from the demands of match-play and therefore limit a players ability to prepare for match-play (32). Players felt the SSG training was more physically and mentally challenging; however, they also found this type of training to be more enjoyable. The coaches also found the SSG intervention increased player adaptability, technical and tactical capabilities greater than traditional training methods.

To the author's knowledge, this is the first study to investigate how to develop Australian football match-play kick proficiency and player adaptability. The SSG intervention group enhanced their kick proficiency by 17%. This may suggest SSG interventions enhance kick proficiency at a faster rate than traditional methods and/or when training interventions more closely simulate match play, the transfer of skill performance from training to match-play will be more prominent. These results are similar to those found by Delextrat and Martinez (33) who conducted a 6 week (twice a week) high-intensity training and SSG basketball intervention on 27 U17 regional level players. The authors found the SSG training intervention resulted in similar aerobic capacity gains, greater defensive agility, greater shooting skills and greater upper body power suggesting this type of training may be appropriate for in-season development of junior players.

The findings of the current study suggest when trying to enhance match-play skill proficiency, the SSG approach may be the more appropriate selection; however, if trying to enhance the technical aspect of the skill (i.e., the fundamentals) then a more traditional approach may be more ideal. Bonney et al. (32) have previously suggested the use of particular skill assessments for desired performance outcomes. For example, on their 5-Level Performance Assessment Model Level-2 (field-based assessments) would assess skill from a technical, isolated and field-based approach whilst Level-5 (match-play) would assess skill from an applied competition approach. The same premise could be applied with the results from this study. When attempting to develop the fundamentals of a skill in a player (e.g., the drop of the ball), traditional approaches may be more appropriate. However, when attempting to enhance match-play skill proficiency in a player, the SSG game intervention appears more effective.

The SSG intervention was found to enhance player adaptability to a greater extent than traditional training methods. This was supported through players executing longer kicks, making quicker decisions, applying more pressure to the opposition and reducing the amount of times skills were executed from a stationary position. Furthermore, both the players and coaches felt the SSG training group were more adaptable and improved their tactical capabilities. In tennis, it has been noted how more experienced tennis players were able to adapt and adjust their visual search strategies to different servers (34). Although visual search strategies were not assessed in this study, the players may have enhanced their search strategies during the intervention stage to enable greater anticipation and movement initiation of the environmental stimuli (e.g., ball or opposition movement). Similar results have been found in futsal where executing passes in a smaller playing area, with shorter time to act, increased player ability when they moved to a larger playing area (35). These results may suggest training on reduced playing areas and having less time to make decisions enhances a players visual search capabilities which makes them more adaptable and enables them to execute more efficient tactical decisions.

Player enjoyment is an important component of the training environment (36). Player questionnaire results indicated the SSG interventions were more enjoyable than the traditional methods. This finding is not unusual as similar results have previously been reported in soccer (37). However, this finding was interesting considering the players also found the SSG interventions were more physically and mentally challenging which can impact player physical and tactical performances (38). The physical fatigue may have been due to the tackling and the repeated changes of direction involved in the SSG, which have been shown to induce high levels of fatigue (39). During traditional training, coaches are cautious of activities that increase the risk of injury (e.g., tackling) and therefore use them sparingly leading to less physical fatigue.

Mental fatigue is an important consideration in Australian football. Previous research in soccer has found mental fatigue to impair running, passing and shooting performances (40, 41). The SSG intervention consisted of limited player numbers within a set playing area, players had to constantly consider their position on the ground, be aware of ball, teammate and opposition movement and provide an option to receive or defend the ball. Combined, these factors may have contributed to the reasons why players felt the SSG training method was more mentally challenging than traditional methods. Overall, these results suggest players experience high levels of physical and mental fatigue during SSGs; however, they enjoy participating in them. Coaches may use this information to implement SSGs when the goal of the session is for the players to experience physical and mental resilience whilst enjoying the session.

The current results confirm the SSG intervention was more successful at increasing player kicking proficiency and player adaptability. The study; however, does contain limitations. The results found in this study were for a small number of amateur senior male players. This group size was; however, deemed appropriate for the study especially as this reflects applied practice were this is a regular group size for an Australian football club training session. Therefore, from an applied perspective, this would be the approximate participant numbers a coach would have if implementing the intervention within their training program. However, it should be noted that further research is recommended to see if the same results would occur for a larger cohort of youth and senior male and female players of different playing abilities. Furthermore, it would be interesting to see how these results may vary if conducted at different times during the session (e.g., at the start), at different stages during the season, how long these adaptations remain for post the final intervention session, what the minimum intervention time frame might be to induce the adaptations found in this study and the performance effect of different SSG interventions. For example, the 5v6 intervention was selected as previous research has found this combination to be similar to those experienced during match-play (5). However, other SSG training interventions (e.g., 7v7) have found players to be under more time pressure when executing a skill whilst less dense playing areas (e.g., 5v5) have found an increase in player physiological effort (7). These recommendations; however, were beyond the scope of this study but provide exciting future research investigations.

## Conclusion

This investigation was the first to compare the effects of a 4-week traditional training and SSG training intervention on Australian football kick proficiency and player adaptability. Only the SSG intervention group improved their kick proficiency and skill adaptability. This group adapted from the pre-test to the post-test by increasing their kick distance, decreasing the amount of time taken before executing a skill, applying more pressure to the opposition and decreasing the amount of times players were stationary when executing a skill. Both the players and coaches found the SSG intervention to be effective at increasing player adaptability, technical capabilities and tactical capabilities in a physically and mentally challenging environment that the players enjoyed.

The results of this study can be used by coaches when designing and implementing training programs. When attempting to increase match-play player adaptability, visual search capabilities, decision-making capabilities and technical and tactical capabilities the SSG intervention appears more suitable. This may be appropriate for the end phase of the pre-season (leading into the start of the season), during the season for specific tactical preparation of an upcoming opponent or during the season where these attributes have declined. However, if the goal of the session is to develop technical aspects of the skill then traditional training implementation may be more effective. This may be more appropriate for the start of pre-season or during the season where a player needs very specific technique refinement to enhance their skill proficiency. Overall, coaches should carefully consider the training session intervention implemented as different training strategies will elicit different player behavioral adaptations.

## Data availability statement

The original contributions presented in the study are included in the article/supplementary material, further inquiries can be directed to the corresponding author.

## Ethics statement

The studies involving human participants were reviewed and approved by the University Human Research Ethics Committee (HRE21-065). The patients/participants provided their written informed consent to participate in this study.

## Author contributions

NB came up with the contextual idea, wrote the article, and completed the data analysis. PL revised the article, co-wrote the article, and assisted with the contextual idea. KB revised the article and assisted with the contextual idea. All authors contributed to the article and approved the submitted version.

## Conflict of interest

The authors declare that the research was conducted in the absence of any commercial or financial relationships that could be construed as a potential conflict of interest.

## Publisher's note

All claims expressed in this article are solely those of the authors and do not necessarily represent those of their affiliated organizations, or those of the publisher, the editors and the reviewers. Any product that may be evaluated in this article, or claim that may be made by its manufacturer, is not guaranteed or endorsed by the publisher.
